# Feasibility, acceptability and preliminary effect of a community-led HIV self-testing model among adolescent girls and young women in Rural Northern Uganda: a quasi-experimental study protocol

**DOI:** 10.1186/s43058-024-00596-7

**Published:** 2024-05-21

**Authors:** Ronald Olum, Elvin H. Geng, Freddy E. Kitutu, Philippa M. Musoke

**Affiliations:** 1 Makerere University School of Public Health, Kampala, Uganda; 2 Division of Infectious Diseases, Department of Medicine, Washington University in St Louis, MO St Louis, USA; 3 Department of Pharmacy, School of Health Sciences, Makerere University, Kampala, Uganda; 4 Department of Pediatrics and Child Health, School of Medicine, Makerere University College of Health Sciences, Kampala, Uganda; 5 Makerere University-John Hopkins University (MU-JHU) Collaboration, Kampala, Uganda

**Keywords:** HIV/AIDS, Self-testing, Adolescent girls, Young women, Sub-saharan Africa

## Abstract

**Background:**

Adolescent girls and young women (AGYW) in sub-Saharan Africa face a disproportionately higher HIV/AIDS burden despite the global decline in incidence. Existing interventions often fail to adequately address their unique social, economic, and cultural challenges, limiting access to essential HIV/AIDS services, including HIV testing. Emerging evidence indicates that HIV self-testing, a user-friendly and confidential method, enhances HIV diagnosis and linkage to care by targeting these barriers. This study aims to assess the feasibility, acceptability, and preliminary impact of a peer-delivered, community-health worker (CHW)-facilitated HIV self-testing intervention for AGYW in Northern Uganda.

**Methods:**

This mixed-methods quasi-experimental implementation science study will employ a three-fold approach. Firstly, we will conduct baseline formative qualitative research with 50 AGYW, 50 parents/partners to AGYW, 30 CHWs, 15 community leaders, and the district health office to inform the design of a peer-delivered CHW-facilitated HIV self-testing intervention tailored to AGYW’s needs in Northern Uganda. Secondly, we will implement a mixed-methods pilot study to assess the intervention’s feasibility and acceptability, involving 415 AGYW, 30 AGYW peer leaders, and 10 CHWs in selected parishes and villages in Omoro district, Northern Uganda. Lastly, we will evaluate the implementation outcomes and preliminary impact of the intervention on HIV self-testing rates and linkage to care by collecting and analyzing quantitative data pre- and post-intervention, laying the groundwork for a future robust randomized controlled trial.

**Discussion:**

Our intervention combines CHWs and peer-led strategies to address the unique challenges of AGYW in Northern Uganda, leveraging community resilience and peer influence. Successful completion of this project will provide a scalable model to be evaluated in a randomized trial and replicated in similar contexts.

**Trial registration number:**

PACTR202404851907736. Registered with the Pan-African Clinical Trials Registry on April 22, 2024.

**Supplementary Information:**

The online version contains supplementary material available at 10.1186/s43058-024-00596-7.

Contributions to the literature

- Despite a global decline in HIV/AIDS incidence, adolescent girls and young women in sub-Saharan Africa still experience a disproportionately high burden of the disease.
- Urgent implementation science research is required to find solutions that enhance access to crucial HIV services for this at-risk population.
- This study aims to develop and assess a community-led HIV self-testing approach for adolescent girls and young women in Northern Uganda, leveraging peer leaders and community health workers.
- Our research seeks to establish the foundation for future robust trials investigating the impact of integrated community-based models on the uptake of HIV services in young people, both locally and internationally.

## Introduction

While there has been significant progress in combatting HIV/AIDS over the past three decades, adolescent girls and young women (AGYW), particularly in sub-Saharan Africa (SSA), remain disproportionately affected, emerging as a high-priority population [1, 2]. In 2022, there were 350,000 new cases of HIV among young people globally, and 60% of these were in AGYW [2]. Every week, of the 4,000 new cases of HIV/AIDS among AGYW worldwide, 77% were in SSA, and they accounted for nearly 63% of all new HIV diagnoses in young people in SSA [2]. In Uganda, of the 19,000 new HIV infections in young people aged 15–24 years, 79% were in AGYW, with the prevalence being almost four times higher than in males [2]. This disparity is majorly attributed to socioeconomic and cultural factors such as gender inequalities, limited education and employment opportunities for girls, and widespread gender-based violence, increasing their vulnerability to HIV [1, 3]. Child, early, and forced marriages, mainly due to poverty, limited access to adolescent-friendly sexual and reproductive health services, fear of stigma and discrimination, and unfair policies and laws in some countries, further contribute to the heightened risk [1, 3, 4].

HIV testing services (HTS) are an essential entry point to the HIV care cascade. By facilitating the diagnosis of HIV and early initiation of antiretroviral therapy (ART), HTS contributes to achieving viral suppression, improving clinical outcomes, and significantly reducing the risk of transmission [2]. Awareness of HIV status can empower individuals to make informed choices about their sexual health, including preventive measures. HTS are also routinely used to track the progression of the HIV epidemic, offering insights into the epidemic projections and the effectiveness of prevention strategies [2]. However, despite the importance of HTS, only 35% of 15–24-year-olds in SSA have ever been tested for HIV [5]. Additionally, only 66% of AGYW living with HIV in SSA are aware of their status, with an estimated 563,000 undiagnosed [5]. In Uganda, HIV testing rates among AGYW range between 43.4% and 84.4% [6–8]. Therefore, conventional health facilities and outreach HTS may not effectively reach all AGYW, especially those at the greatest risk. Barriers such as costs related to accessing HTS, the stigma associated with seeking such services, deeply rooted cultural and religious beliefs, and laws or policies requiring parental consent for HTS often hinder access [7, 9–11], especially in rural settings. Consequently, there is an urgent need for innovative, people-centered solutions to address these barriers [2, 12].

HIV self-testing (HIVST) has emerged over the past decade as an essential intervention to mitigate the challenges of conventional HTS. HIVST offers two modalities, blood-based and oral-fluid-based tests, with high sensitivity and specificity [13]. It has also been shown to be effective in diagnosing HIV in trials and real-world settings [14, 15]. HIVST is more preferred, acceptable, and feasible among AGYW, including in SSA [16–18]. In a fishing community in central Uganda, 74.2% of AGYW had heard about oral HIVST, and all were willing to use the kit if made freely available [19], while 93% of AGYW at a Ugandan tertiary institution were ready to use HIVST kits [20]. Despite robust evidence of its efficacy and tangible success in real-world settings, its uptake remains low in many high-risk populations [16]. Barriers to the implementation of HIVST include low awareness among AGYW, limited accessibility, high costs, stigma, and indirect obstacles like intimate partner violence [16]. Moreover, while HIVST offers initial diagnosis, challenges persist in linking those who test positive to timely and appropriate care, potentially leaving gaps in the treatment and management continuum [14].

Strategies like integration with existing sexual and reproductive health services [21], school-based distributions [22, 23], digital initiatives [24], and community-based models [19, 25] are potential options for increasing HIVST uptake among AGYW [14, 16]. Peer-led models have demonstrated promising outcomes, evidenced by studies in Kenya [26], Zimbabwe [27], South Africa [17], and Nigeria [28]. In a Ugandan fishing community, peer leaders distributed 99.3% of HIVST kits to AGYW, and 98.2% used the kit [19, 29]. In Kampala, all the 30 AGYW surveyed in a PrEP clinic embraced peer-delivered HIVST, citing its convenience, travel cost savings, reduced clinic waiting times, and stigma-free environment [30]. While CHW-led models have shown promise in delivering HIVST kits among AGYW in places like Malawi [25, 31, 32] and Zimbabwe [33, 34], a significant research gap among AGYW exists in Uganda. A recent CHW-led HIVST strategy targeting men in Uganda enhanced HIV testing rates, disclosure, and ART initiation [35, 36].

Recently, there have been calls from UNAIDS and an increasing recognition in the scientific community to embrace community-led initiatives in the response to HIV [37, 38]. To our knowledge, there are no published studies in Uganda evaluating the community-led HIVST model for AGYW in post-war Northern Uganda despite its unique sociocultural context. Our study aims to develop and evaluate the acceptability, feasibility, and preliminary effect of a peer-delivered and CHW-facilitated HIVST model among AGYW in rural Northern Uganda.

## Conceptual framework

**Fig. 1:**
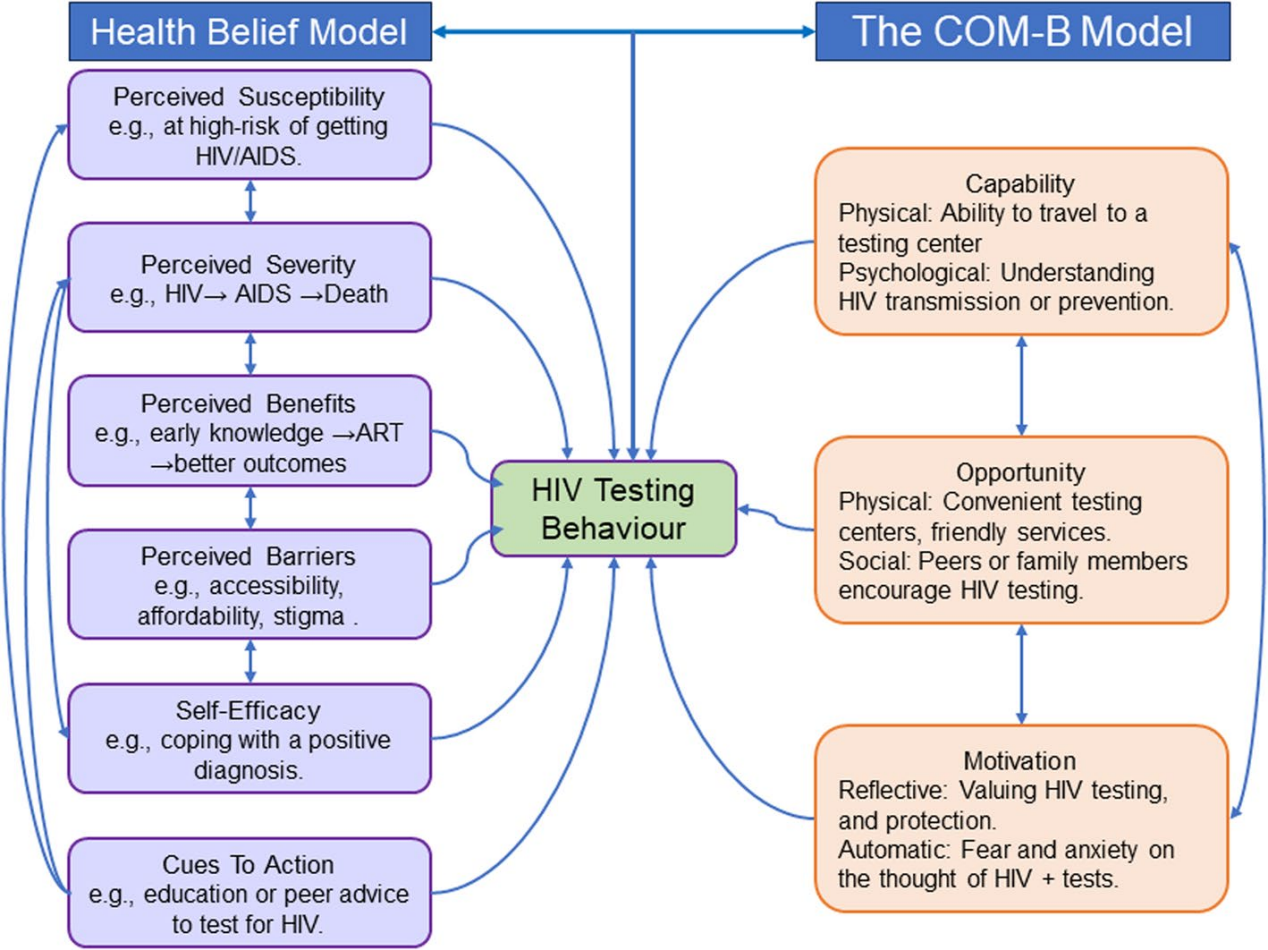
An illustration of the facilitators and barriers to HIV testing among adolescent girls and young women using the COM-B and the Health-Belief Models

The formative research phase of our intervention will be guided by a combination of two models: the Capability, Opportunity, and Motivation (COM-B) model [39] and the Health Belief Model (HBM) [40], providing a multifaceted lens through which to explore and understand the complexities of HIV testing among AGYW in Northern Uganda. The COM-B Model postulates that behavior (B) is a product of the interaction between Capability (C), Opportunity (O), and Motivation (M). In the context of our formative research, the COM-B Model will enable us to determine the various components that influence AGYW’s HIV testing behaviors and how these might be targeted in our intervention (Fig. 1). The HBM posits that individuals are more likely to engage in health-promoting behavior like HIV testing if they perceive themselves to be susceptible to a health problem, believe it has serious consequences, believe taking a specific action would reduce their susceptibility to or severity of the health problem, and believe the benefits of taking the action outweigh the costs or risks. The HBM will guide the exploration of beliefs and perceptions related to HIV and HIV testing, including self-testing, thereby informing strategies to enhance perceived benefits and minimize perceived barriers in our intervention.

**Fig. 2:**
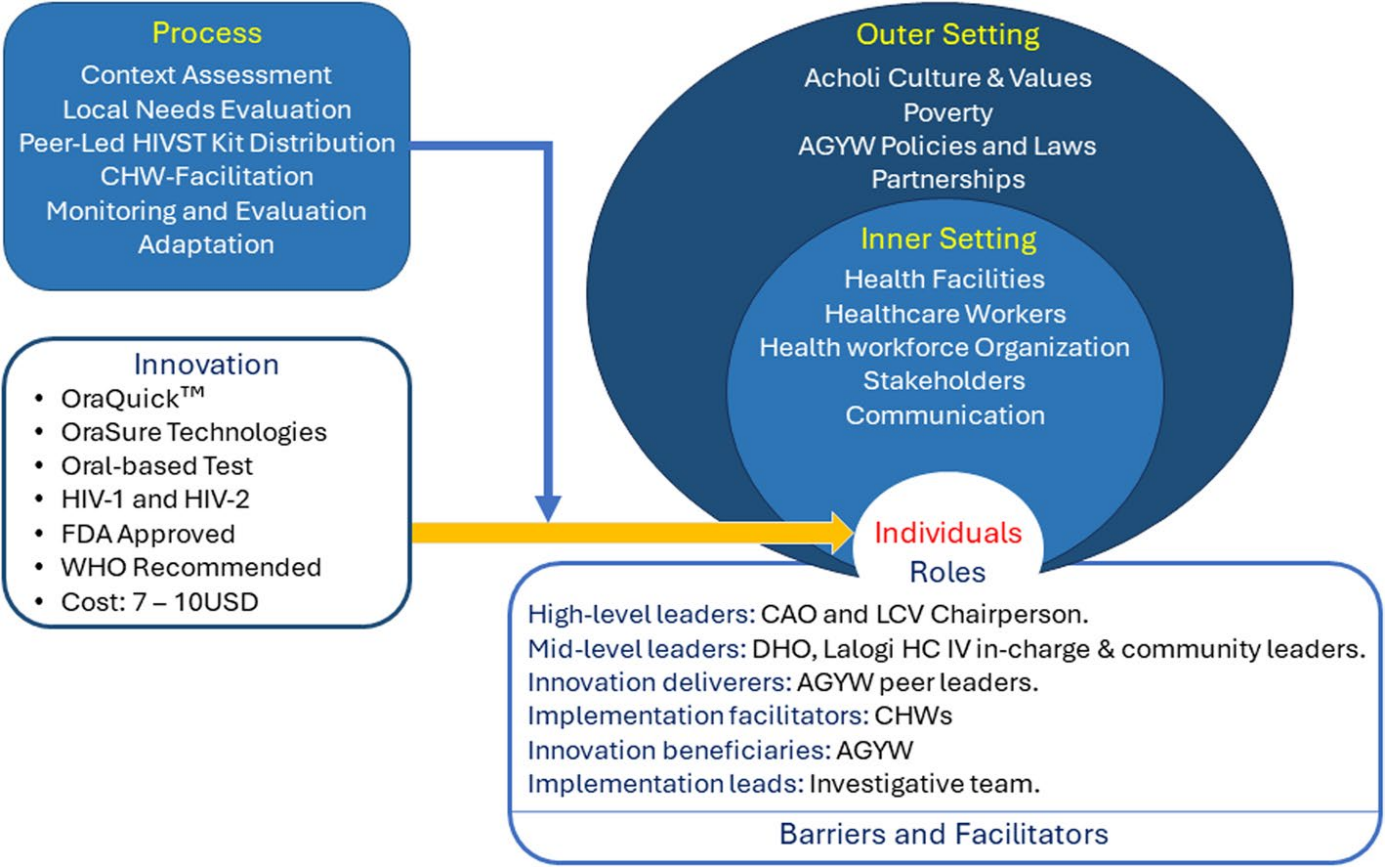
The updated Consolidated Framework for Implementation Research showing factors that may influence the implementation of a peer-led CHW-facilitated HIVST among AGYW in Northern Uganda (Image adapted from The Center for Implementation [42])

The updated Consolidated Framework for Implementation Research (CFIR) [41] will be used to facilitate a comprehensive understanding of the multifaceted factors influencing the implementation of CHW-facilitated peer-led HIVST intervention among AGYW in rural Northern Uganda (Fig. 2). The **innovation**, OraQuick, manufactured by OraSure Technologies, is a rapid HIV self-test that uses an oral swab to detect antibodies to HIV-1 and HIV-2. Its efficacy has been demonstrated in numerous trials and real-world settings, showcasing its potential as an effective tool for HIV screening. The sensitivity of OraQuick ranges from 97.7 − 99.6% and specificity from 96.8 − 99.9%, emphasizing its accuracy and reliability. The test was approved by the U.S. Food and Drug Administration (FDA) in 2012 and is listed among accepted pre-qualified in vitro diagnostics by the World Health Organization. Evidence suggests that it is simple to use by AGYW with minimal or no supervision and returns results within 20 min. In the Ugandan retail pharmacies, it costs between 7 USD – 10 USD.

In the **outer setting**, the sociocultural values of the local ethnic groups in this region and the economic circumstances will significantly influence the acceptability and feasibility of our intervention. Northern Uganda is mainly inhabited by two ethnic groups - Acholi and Lango - who belong to the Luo origin and dialect and share many similarities in language and culture. The Acholi and Lango occupy the Acholi and Lango sub-regions, respectively. In addition to social and cultural values, partnership with the local leaders and adherence to policies and laws while respecting local traditions will facilitate the smooth delivery of the intervention. Anticipated critical events include inconclusive results, emotional and psychological impact on individuals receiving positive results, confidentiality breaches, stigma, discrimination, and navigating ethical dilemmas related to disclosure, particularly in AGYW with guardians or partners. The implementation team shall undergo didactic training before and during the intervention to mitigate such events.

The **inner setting**, which includes the physical infrastructure (the nearby health facilities in each sub-country), work infrastructure (health workers, including CHWs), and the organization of the community, is essential to the success of the intervention. Ensuring that the intervention aligns with the norms and values of the CHWs and the community, and prioritizing communication with stakeholders, will be critical for effectively navigating the complex intervention environment. The target **individuals** in this study are AGYW, aged 15–24 years, in Omoro district, and they are the innovation recipients. The high-level leaders include the local government officials of Omoro district, particularly the district Chief Administrative Officer and the District Chairperson, whose approval will be sought before project implementation. The District Health Officer of the district and Lalogi Health Sub-District in charge are mid-level leaders and will provide technical support and guidance in mobilizing health workers needed for confirmatory testing, linkage to care, ART initiation, and follow-up. The village local council chairpersons, who are also mid-level leaders, will facilitate community entry and mobilization. The CHWs are the implementation facilitators, while the AGYW peer leaders are the innovation deliverers. The investigative team will be the implementation leads. The formative research will provide more insights into the characteristics of the individuals regarding their capability, opportunities, motivation, and needs.

Finally, the **implementation process**, as previously described, will involve formative research to assess the needs and context, adapting and tailoring strategies to the local context, and teaming up AGYW leaders with CHWs. This will, in turn, strengthen the already existing working relationships between CHWs and the nearest health facilities for effective linkage to care.

The Social Cognitive Theory (SCT), an essential framework for understanding, predicting, and altering human behaviour, shall be used to describe the theory of change while evaluating the preliminary effects of the intervention [43]. The SCT postulates that human learning and behaviour are products of a dynamic interplay between personal, behavioral, and environmental factors. It emphasizes the role of observational learning, motivational processes, attention, retention, self-efficacy, and outcome expectations in behavior development and maintenance. In our context, AGYW are more likely to accept HIVST when promoted, distributed, trained, counselled, and facilitated by their peers who live within their communities who are relatable to them. Seeing or hearing feedback that other peers are also engaging in HIVST can reduce the fear and stigma associated with the testing process, promoting its adoption. Feedback from peers about the benefits of HIVST, like early detection, better outcomes, and peace of mind that comes with knowing their status, they may be more likely to accept HIVST and linkage to care. Furthermore, using AGYW-peer-leaders to distribute HIVST kits recognizes young people as important stakeholders in their own health, capturing the attention of fellow AGYW better, improving sustainability, and promoting community resilience.

## Methods and materials

### Aims

The overall aim of this study is to establish a sustainable, community-based HIV self-testing model that effectively increases HIV rates and linkage to care among AGYW in Northern Uganda. The specific aims are:

1. To develop a peer-delivered CHW-facilitated HIV self-testing model tailored to the needs of AGYW in Northern Uganda.
2. To evaluate the feasibility and acceptability of the peer-delivered CHW-facilitated HIVST intervention in Northern Uganda.
3. To assess the preliminary impact of the peer-delivered CHW-facilitated HIVST model on HIV self-testing rates and linkage to care among AGYW.

### Study design

This will be a quasi-experimental mixed-methods implementation study utilizing both qualitative and quantitative methods. This protocol adheres to the Standard Protocol Items: Recommendations for Interventional Trials (SPIRIT) statement [44] and the Template for Intervention Description and Replication (TIDieR) guidance [45]. Filled copies of the checklists are attached as Supplementary File 2 and Supplementary File 3, respectively.

### Study setting

This study will be conducted in Omoro district, located south of Gulu and North of Kampala, Uganda’s capital city. Omoro is one of the districts affected most by over two decades of civil conflict in Northern Uganda, which led to mass destruction of property, displacement, and loss of lives. It is also among the poorest districts in the region and the country, with a GDP per capita of 183 USD [46]. Lalogi Health Center IV is the district’s largest public health facility, with 5 Health Center IIIs and 15 Health Center IIs. CHWs (also known as village health teams) provide the first line of healthcare at the village level, mainly preventive and referral services. Omoro is divided into Tochi and Omoro counties and has twelve sub-counties.

### Study population

The primary target population for this study is adolescent girls and young women (AGYW) aged 15–24 years living in Omoro district of Northern Uganda. The secondary target population are community health workers (village health team members) and the local council leaders in Omoro district.

### Sample size and sampling techniques

A multistage random sampling method will be employed to select study participants representative of the district’s population. Initially, we will randomly select five sub-counties from the available twelve in the district. One parish will then be selected randomly from each of the five sub-counties as study sites. For the baseline qualitative focus group discussions (FGDs), a total of 50 matched pairs of AGYW and their parents or spouses (for AGYW in marriage or union) will be selected using purposive sampling across five parishes. We will identify ten homesteads in each parish in collaboration with local council leaders, aiming to ensure a diverse representation of geographical, educational, and socioeconomic backgrounds. This number is based on evidence suggesting that saturation in qualitative studies often occurs by the twelfth interview, with minimal new information beyond 20–30 interviews [47]. A larger sample size was chosen to capture a diverse range of experiences and perspectives within this group from all the five selected parishes. Recognizing the iterative nature of the qualitative methods, we will also implement adaptive sampling strategies, allowing for the inclusion of additional participants or specific subgroups as new themes emerge until no new information is obtained and saturation is reached. Thirty CHWs and fifteen community leaders (local council chairpersons I, II, and IIIs) will be purposively selected, in line with qualitative research methodologies that recommend smaller, focused samples for specialized groups [48], to adequately explore their unique perspectives on the HIV self-testing model.

The sample size for the quantitative pre- and post-intervention quantitative survey among AGYW was calculated using Epi Info StatCalc for population surveys and was powered with acceptability as the primary outcome variable. As of the last national census, females make up 51% of the population in Omoro, leading to an estimated population of 16,383 AGYWs. At an average population growth rate of 3.2% between 2014 and 2022, the estimated population of AGYW in Omoro district is 21,753 by the end of 2023. To estimate the acceptability of HIVST among AGYW with an estimated population size of 21,753, expected acceptability of 50% since no previous studies have been conducted in this region, a margin of error of ± 5% at a 95% confidence level, and a design effect of 1.0, we determined that a sample size of at least 377 AGYW is required. To cater for non-response and loss-to-follow-up, an additional 10% of the sample size will be added, leading to a final sample size of 415 AGYW. The study participants will be selected through systematic random sampling, depending on the number of households obtained from the local council leaders. First, we will obtain the number of potentially eligible homesteads from the local council chairpersons in the selected parishes. We will then calculate the sampling interval (k) by dividing the total number of eligible homesteads by the desired sample size. Starting with a random point between 1 and k, we will select every kth person from the list for participation. In circumstances where the household members are not around during the interview or decline consent, we shall consider the next immediate household and then resume the sampling interval.

### Selection criteria

All adolescent girls and young women aged 15–24 years who have lived in the selected parishes for at least three months and do not plan to leave their respective villages in the next one year will be eligible to participate in this study, after providing informed consent. Eligible peer leaders will be adolescent girls and young women aged 15–24 years, who have lived in their communities for at least six months with no intention to migrate within three months with basic English and Luo comprehension, good communication skills, and are viewed as role models with integrity in their communities. All AGYW with a confirmed diagnosis of HIV/AIDS and/or on antiretroviral therapy prior to this study will be excluded.

### Study procedures

**Fig. 3:**
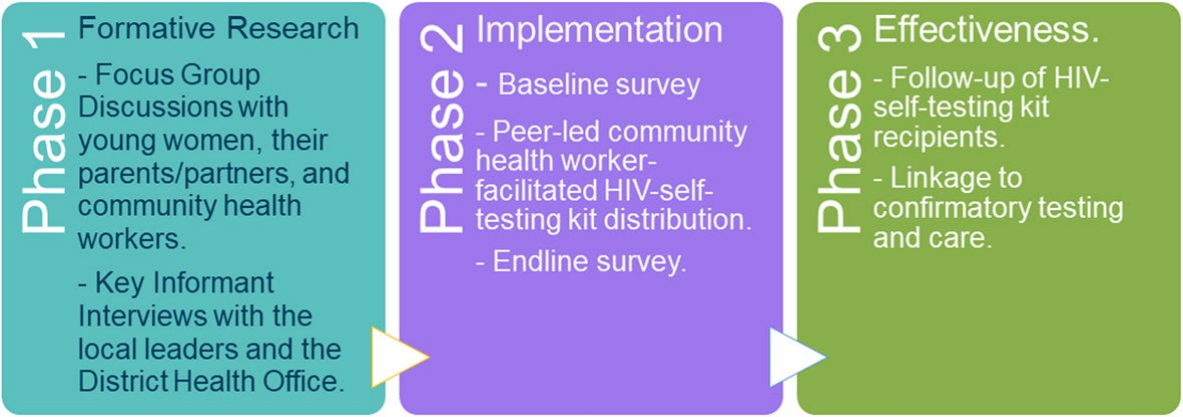
A schematic description of the study showing the three phases of implementation research

The key phases and procedures of this study are summarized in Fig. 3 below.

### Formative phase

We will conduct a qualitative exploratory study among AGYW, CHWs, community leaders, and parents and/or spouses of AGYW from the five selected parishes before the intervention. A total of 50 AGYW, 50 parents and/or spouses for married AYGW, and 30 CHWs from five parishes will participate in 5 (FGDs), each with a composition of 10 participants per FGD. This is in line with the literature, which suggests that for FGDs, 4–6 groups are typically sufficient to reach saturation in homogeneous populations, with each group consisting of 6–10 participants [49]. Additionally, 15 community leaders from the five parishes will be selected purposively for in-depth interviews (IDIs). The FGDs and IDIs will explore the participants’ knowledge and perceptions of HIV, experiences with HIV testing services, and preferences regarding peer-led and CHW-facilitated HIVST models. All the FGDs and KIIs will be conducted in Luo and will be recorded. We will also conduct one key-informant interview (KII) with the district health office to explore the landscape surrounding the provision of HIV services in the district, as well as to map potential stakeholders and facilitators of success. Each interview will be moderated/conducted by two research assistants with experience in qualitative research and fluency in both languages after comprehensive training.

The findings from our formative research will lay a crucial foundation for designing the intervention and further adaptation. We shall utilize on-the-go adaptation strategies previously described by Elvin Geng and colleagues to tailor the intervention strategy [50]. The adaptations may involve refining communication messages, adjusting delivery methods, or redefining the roles of peer leaders and CHWs.

### Baseline pre-intervention survey

Before the intervention, 415 AGYW, aged 15–24 years, who have lived in their communities for at least three months, will be selected from the five parishes using systematic random sampling at a household level and administered a baseline semi-structured survey. Sociodemographic data, knowledge, perceptions, and practices on HIV and HTS, including HIVST, will be collected. The questionnaire will also evaluate the feasibility, acceptability, and appropriateness of HIVST and the peer-delivered CHW-facilitated HIVST model.

### Implementation strategy

With the guidance of the community leaders and ten purposively selected CHWs, we will identify 30 AGYWs from the five parishes to serve as peer leaders. The peer leaders and CHWs will undergo comprehensive training on the project, their roles, and the technical aspects of HIV/AIDS, HIVST procedures, basic counselling, and linkage to care. We will also train them in community mobilisation, communication, and engagement. The CHWs will allocate oral HIVST kits (OraQuick™) to peer leaders, who will distribute them to AGYW selected by systematic random sampling in their respective villages over three months. Each AGYW peer leader will then be instructed to distribute HIVST kits to 13 of the selected AGYW from their villages within three months. Adolescent girls and young women who desire to receive additional HIV self-testing kits for their partners/spouses will also be provided.

The kit will also contain informational material in English and Acholi, containing the testing instructions, a confidential results card, and mobile contacts of CHWs, counsellors, and health facility focal persons to support confirmatory testing and ART initiation for those who test positive. Distributions will take place through various strategies, including door-to-door, community group meetings, and at other preferred locations. They will be carried out at different times, including weekends and evenings, to maximise accessibility for AGYW with varied schedules. The peer leaders will demonstrate the HIVST kit usage to the AGYW and remind them to perform the test for up to two weeks following the initial distribution.

The CHWs will then follow up with all the AGYWs who conduct the HIV self-test, seeking their consent for voluntary disclosure of test results and facilitating linkage to confirmatory testing and ART initiation. CHWs will also support community entry, manage logistics, oversee HIVST distribution by peer leaders, and provide continuous support.

### Post-intervention survey

To explore post-intervention acceptability, appropriateness, feasibility, experiences, perceived barriers, facilitators, and overall perceptions toward the peer-delivered and CHW-facilitated HIVST model, we will conduct a follow-up semi-structured survey with all the study participants using a semi-structured questionnaire. Additionally, we shall conduct nine FGDs: five among AGYW who were scheduled to receive the intervention, three among AGYW peer leaders, and one among CHWs. Each FGD will consist of ten participants. We will also track and record the number and trends of HIVST kits distributed through the peer leaders and the CHWs to assess feasibility. Fidelity will be assessed using observational checklists and qualitative feedback from the AGYW, peer-leaders, and the CHWs. We will also track and record all costs associated with the implementation in record-keeping books for an economic evaluation of the model.

### Post-intervention follow-up

We will follow up with all the AGYW who received HIVST kits for up to one month to assess for self-reported use of the HIVST kits. AGYW will also be asked to share their test results with the study team anonymously using confidential cards included in the HIVST kit package to evaluate the positivity rate. We will also track the number of confirmatory tests, linkages to care, and ART initiation among AGYW who test positive using data from the CHWs and the health facility contact persons.

### Study outcomes

The primary outcomes (acceptability and feasibility) and the secondary outcomes (appropriateness, fidelity, and cost) are derived from the Proctor Framework, with details of measurements in Table 1 [51]. Acceptability is defined as the perception by the stakeholders (AGYW, peer leaders, and CHWs) that the model is agreeable or satisfactory. Feasibility is defined as the degree to which the HIVST is successfully distributed through the peer-delivered CHW-facilitated model. Preliminary effectiveness will be defined as the proportions of AGYW who perform an HIV test within one month of HIVST kit reception, are linked for confirmatory testing, and ART initiation.

**Table 1:** Implementation outcomes to be evaluated in this study (adopted from the Proctor Implementation Framework)

Outcome	Measurements
Acceptability	• The proportion of AGYW who report the peer-delivered CHW-facilitated HIVST model as agreeable and satisfactory before and after the implementation. • The proportion of peer leaders and CHWs who find the delivery process acceptable. • Qualitative feedback from AGYW, peer leaders, and CHWs.
Feasibility	• The proportion of planned HIVST kit distribution that will successfully be carried out. • Qualitative feedback from CHWs and peer leaders regarding challenges or barriers.
Appropriateness	• The proportion of stakeholders (AGYW, CHWs, community members) who perceive HIVST as a relevant and fitting solution for HIV testing challenges.
Fidelity	• The proportion of CHWs and AGYW peer-leaders who correctly perform all the planned activities during the HIVST distribution. • The proportion of AGYW who will receive all components as planned.
Cost	• Financial and economic costs associated with HIVST kits procurement, training, and distribution (including reimbursing peer leaders and CHWs). • Cost comparison to potential long-term savings or benefits.

### Data management and analysis

The FGDs and KIIs recordings will be transcribed verbatim, translated to English, and exported to *Atlas.ti* for thematic content analysis to inform the development and adaptation of intervention. Transcriptions from the FGDs and KIIs will be thematically analysed to identify themes related to implementation outcomes, experiences, barriers, and facilitators of peer-delivered CHW-facilitated HIVST. Upon importing the transcribed data into Atlas.ti, we will initially conduct a thorough reading to familiarize ourselves with the content, marking significant and recurrent themes related to barriers and facilitators to HIV and HIVST among AGYW in Northern Uganda. Coding will be performed iteratively, with initial codes generated from the data being grouped into broader themes and sub-themes that capture the nuances of participants’ experiences and perceptions.

Quantitative data from the semi-structured questionnaires and observational checklists will be collected by trained research assistants using RedCap™ deployed into a tablet, uploaded on secure cloud storage, and exported to STATA 18.0 and R Software for cleaning, coding, and analysis. At the univariate level, we will describe demographic characteristics and implementation outcomes using frequencies and percentages for categorical variables, means and standard deviation for normally distributed continuous variables, and median and interquartile range for non-parametric distribution. Factors associated with acceptability will be assessed using multilevel logistic regression models, adjusting for confounders. Factors to be included in the model will be selected from the literature review guided by the COM-B, HBM, and CFIR theoretical frameworks and expert opinion. We will also evaluate changes in the implementation outcomes before and after the intervention using paired t-test, Mann-Whiney U test, and McNemar’s Test, appropriately. A *p*-value < 0.05 will be considered statistically significant. We will estimate the overall cost of implementing the intervention using the data recorded in the financial books. The costs per HIV test kit distributed, HIV test performed, and HIV diagnosis will be calculated to facilitate a cost-benefit analysis of the model.

The preliminary effectiveness outcomes that will be assessed include HIV testing uptake, confirmatory HIV testing, and linkage to care. Because of the modest sample size powered to assess acceptability, we will provide descriptive findings on the proportion of AGYW that performed HIV testing after receiving HIVST kits, the proportion that tested positive on confirmatory HIV testing, and the number of AGYW that were linked to care. These results will guide future large-scale controlled trials on the effectiveness of the peer-led CHW-facilitated HIVST model.

## Discussion

There is a critical need for implementation strategies that can overcome important contextual barriers that prevent AGYW, especially in SSA, from accessing essential HIV/AIDS services, including HIV testing. Our proposed intervention innovatively combines peer and CHW-led HIVST models among AGYW in Uganda, addressing their distinct cultural, social, and economic challenges. It harnesses the established individual strengths of both models in HIV/AIDS care and their application in other health conditions [16]. CHWs represent the first-level health facility at the village level and have been pivotal in the HIV care cascade [52]. Peer leaders, supported by studies showing their effectiveness in promoting health, resonate strongly with AGYW, providing a comprehensive and relatable support system [53]. Leveraging the pre-existing reach and trust in CHWs and combining it with peer-led strategies can foster an environment where AGYW feel more comfortable engaging with HIVST. We also attempt to address linkage to care, a widely recognised limitation of HIVST, by strengthening the existing CHW referral system to enhance access to confirmatory testing and early ART initiation among AGYW.

Additionally, our model fosters community resilience by empowering existing local structures, which can potentially facilitate adoption, penetration, scalability, and sustainability beyond the study’s duration. Engaging AGYW as peer leaders empowers them to take active roles in their health, especially in gender-normative contexts where women traditionally have limited agency, like traditional Acholi settings. Our study has an indirect potential to reduce the high adolescent pregnancy rates in the region by promoting healthier sexual relationships. Successful completion of this study will yield a comprehensive, contextually relevant, and scalable intervention model that can be evaluated in a randomized trial and replicated in similar settings. By increasing the rate of HIV self-testing and improving linkage to care, we can reduce the prevalence of undiagnosed and untreated HIV among adolescent girls and young women in Northern Uganda, thereby contributing to improved health outcomes for this population, reducing the transmission of HIV, and moving closer to ending the HIV/AIDS epidemic in Uganda and beyond. By enhancing HIV testing coverage in such underserved areas, we strive to contribute to achieving the first target of UNAIDS 95-95-95 goal by 2030. Finally, our findings will lay the groundwork for subsequent trials investigating the effect of integrated community-based models on AGYW’s clinical outcomes locally and globally.

We anticipate difficulty accessing study participants during school and working hours, which will be mitigated by conducting interviews during holidays, weekends, and evenings. Secondly, due to cultural norms related to decision-making and agency in the region, some participants may face gender-based violence as a result of taking part in this study. To mitigate this, we will informally seek permission from the household heads before the interviews and written informed consent from the participants. The potential mental health impact of getting a positive HIV result after a self-test will be mitigated through HIV counseling before the tests, contacts to counselors included in the testing kits, and follow-up by CHWs for voluntary disclosure, confirmatory testing, and linkage to care.

## Conclusion

Community-led HIV self-testing models leveraging peer leaders and community health workers and specifically tailored to the needs and circumstances of young women could potentially increase HIV testing rates, diagnosis, and linkage to care. By reducing significant barriers to accessing HIV testing and care, such as discrimination, stigma, and costs, this model may be more sustainable and cost-effective, warranting further research.

### Supplementary Information

Supplementary Material 1.

Supplementary Material 2.

Supplementary Material 3.

## Data Availability

All data and materials from this study will be available upon reasonable request from the corresponding author (Dr. Ronald Olum) and relevant institutional approval. The findings will be shared with the community and relevant stakeholders through briefs, meetings, media talk shows, and other events, as well as with the scientific community in a peer-reviewed journal and scientific conferences.

## Abbreviations

AGYW: Adolescent girls and young women
ART: Antiretroviral therapy
CFIR: Consolidated framework for implementation research
CHWs: Community health workers
COM-B: Capability, opportunity and motivation
FGD: Focus group discussion
HBM: Health-belief model
HIVST: HIV self-testing
HTS: HIV testing services
KII: Key-informant interviews
SCT: Social cognitive theory
SSA: Sub-Saharan Africa
UNAIDS: Joint United Nations Programme on HIV/AIDS
