# Supplementary material for: Feasibility, acceptability and preliminary effect of a community-led HIV self-testing model among adolescent girls and young women in Rural Northern Uganda: a quasi-experimental study protocol
Source: Implement Sci Commun. 2024 May 21;5:56. doi: 10.1186/s43058-024-00596-7 (PMC11110295; doi:10.1186/s43058-024-00596-7)
Supplement: Supplementary file 1 — Supplementary Material 1. [file 43058_2024_596_MOESM1_ESM.pdf]

CONSENT FORM – ADOLESCENT GIRLS AND YOUNG WOMEN

Feasibility, Acceptability and Preliminary Effect of a Community-Led HIV Self-Testing Intervention Among Adolescent Girls and Young Women in Rural Northern Uganda.

Background and rationale for the study:

Adolescent girls and young women in low-resource settings like rural Northern Uganda face a disproportionately high HIV/AIDS burden, even when there has been success in reducing the number worldwide. Access to essential HIV/AIDS services is sometimes difficult for young women because existing interventions may not address their unique social, economic, and cultural challenges. HIV self-testing is a private and simple way for individuals to find out their HIV status. This approach is promising because it can help overcome barriers to getting tested and accessing health care.

Purpose:

We want to find out if a new approach, where friends help each other and community health workers guide them, can make HIV self-testing work well for young women in Northern Uganda. We are looking to see if this idea is practical if young women are open to trying it, and how it might start making a difference in our community.

Who will participate in this study?

This study will include adolescent girls and young women, their parents or spouses (for married young women), community health workers, local leaders, and the district health officer in Omoro district.

Study procedures.

If you agree to participate in this study, you will be asked to respond to a questionnaire with 70 questions. The questionnaire covers topics such as your background history, HIV-related history and perceptions, and your thoughts on the proposed method of HIV self-testing. A research assistant will administer the questionnaire to the participant. The research assistant will also help you if you have questions or difficulties answering them. This process is expected to take between 40 to 60 minutes.

Risks/Discomforts:

Joining this study poses minimal risk, not much different from what you experience every day or during regular check-ups. However, talking about HIV, your past HIV tests, and this new way of testing with help from friends and health workers might bring up strong feelings or remind you of tough times. We have a caring team ready to help you if this happens, and they can connect you with more support if you need it.

Benefits:

By being part of this study, you are giving us essential information that will help us figure out the best way to introduce HIV self-testing to young women in Northern Uganda. This could make it easier for more people to test themselves for HIV and get the help they need. Your involvement not only helps you understand and deal with HIV better but also helps everyone by improving how we fight against HIV/AIDS in places like ours.

Compensation for participation in the study:

For participating in this study, you will receive [redacted] Uganda shillings for the time you spend with us and [redacted] Uganda shillings to cover travel costs. If anything goes wrong and you get hurt while taking part in the study, we will immediately link you with appropriate medical services at the nearest healthcare facility.

Confidentiality:

The results of this study will be used for research and policy purposes only. The questionnaires have been designed so that no information can be used to recognize the identity of the participants. Your details, such as name, employee number, registration number, and address, are not required anywhere in the data collection tool. Paperwork shall be kept under lock and key, whereas computer data shall be protected with passwords. Access to data is limited to the investigators only. Makerere University of Health Sciences Research Ethics Committee and Uganda National Council for Science and Technology may also have access to the data as required by the law.

Statement of voluntariness:

Participation in this proposed study is voluntary. Participants may join on their own free will and have the right to withdraw from the study without penalty.

Dissemination of results:

Research participants will receive feedback on the study's findings and progress, and any new information that affects the study or data that has clinical relevance to research participants (including incidental findings) will be made available to them and their healthcare providers.

Questions.

If you have any questions about the study, please contact the study staff interviewing you or the Principal Investigator.

Dr. Ronald Olum; Email: olum.ronald@gmail.com, Mobile: +256775512540.

For any queries related to the study participant's rights, kindly address them to the Chairperson of Makerere University School of Health Sciences Research Ethics Committee,

Dr. Paul Kutyabami; Mobile: +256 0772 404 970, Email: paulkutyabami72@gmail.com

STATEMENT OF CONSENT

.... has described to me what is going to be done, the risks, the benefits involved and my rights regarding this study. I understand that my decision to participate in this study will not alter my usual medical care. In the use of this information, my identity will be concealed. I am aware that I may withdraw at any time. I understand that by signing this form, I do not waive any of my legal rights but merely indicate that I have been informed about the research study in which I am voluntarily agreeing to participate. A copy of this form will be provided to me.

Name of the participant ……………………………………………………………………………….

Signature/thumbprint of participant …………………………………………Date……………………….

Name of parent/guardian for minors (If applicable) ……………………..…………………..………

Signature/thumbprint of parent/guardian ………………………………… Date……..………………..

Name of the witness (If applicable) ………………..…………..…………..……………………………

Signature/thumbprint of the witness …………………………………………Date……………..………

Name of interviewer …………………………………………………………………………..………….

Signature of interviewer …………………………………….……………………Date …………………….
